# RETRACTION: The Effect of the Multiple Arterial Grafts Compared with Single Arterial Graft for Coronary Artery Bypass Grafting on Sternal Wound Complications: A Meta‐Analysis

**DOI:** 10.1111/iwj.70214

**Published:** 2025-02-11

## Abstract

**RETRACTION**: YangX. , FuJ. , and ZhangS. , “The Effect of the Multiple Arterial Grafts Compared with Single Arterial Graft for Coronary Artery Bypass Grafting on Sternal Wound Complications: A Meta‐Analysis,” International Wound Journal 20, no. 8 (2023): 3249–3254, 10.1111/iwj.14204.37132096 PMC10502291

The above article, published online on 02 May 2023, in Wiley Online Library (http://onlinelibrary.wiley.com/), has been retracted by agreement between the journal Editor in Chief, Professor Keith Harding; and John Wiley & Sons Ltd. Following an investigation by the publisher, all parties have concluded that this article was accepted solely on the basis of a compromised peer review process. The editors have therefore decided to retract the article. The authors did not respond to our notice regarding the retraction.

**RETRACTION**: X. Yang , J. Fu , and S. Zhang , “The Effect of the Multiple Arterial Grafts Compared with Single Arterial Graft for Coronary Artery Bypass Grafting on Sternal Wound Complications: A Meta‐Analysis,” International Wound Journal 20, no. 8 (2023): 3249–3254, 10.1111/iwj.14204.37132096 PMC10502291

The above article, published online on 02 May 2023, in Wiley Online Library (http://onlinelibrary.wiley.com/), has been retracted by agreement between the journal Editor in Chief, Professor Keith Harding; and John Wiley & Sons Ltd. Following an investigation by the publisher, all parties have concluded that this article was accepted solely on the basis of a compromised peer review process. The editors have therefore decided to retract the article. The authors did not respond to our notice regarding the retraction.

